# Genetic diversity and recombination in natural populations of the nematode-trapping fungus *Arthrobotrys oligospora* from China

**DOI:** 10.1002/ece3.450

**Published:** 2013-01-07

**Authors:** Ying Zhang, Min Qiao, Jianping Xu, Yang Cao, Ke-Qin Zhang, Ze-Fen Yu

**Affiliations:** 1 Laboratory for Conservation and Utilization of Bio-resources, and Key Laboratory for Microbial Resources of the Ministry of Education, Yunnan UniversityKunming, Yunnan, 650091, China; 2 Department of Biology, McMaster University1280 Main Street West, Hamilton, Ontario, Canada, L8S 4K1

**Keywords:** Ecological genetics, fungi, population ecology, population genetics – empirical

## Abstract

Nematophagous fungi can trap and capture nematodes and other small invertebrates. This unique ability has made them ideal organisms from which to develop biological control agents against plant- and animal-parasitic nematodes. However, effective application of biocontrol agents in the field requires a comprehensive understanding about the ecology and population genetics of the nematophagous fungi in natural environments. Here, we genotyped 228 strains of the nematode-trapping fungus *Arthrobotrys oligospora* using 12 single nucleotide polymorphic markers located on eight random DNA fragments. The strains were from different ecological niches and geographical regions from China. Our analyses identified that ecological niche separations contributed significantly, whereas geographic separation contributed relatively little to the overall genetic variation in our samples of *A. oligospora*. Interestingly, populations from stressful environments seemed to be more variable and showed more evidence for recombination than those from benign environments at the same geographic areas. We discussed the implications of our results to the conservation and biocontrol application of *A. oligospora* in agriculture and forestry.

## Introduction

Fungi possess diverse strategies to obtain nutrients, as saprobes, pathogens (of plant and animal hosts), and symbionts (with other microbes, plants or animals). These strategies enable fungi to colonize a broad range of habitats on plants and animals (including humans), in agricultural and forest soils, and in aquatic environments. They can also be found in sites contaminated by heavy metals and grow on hypersaline microbial mats (Cantrell and Baez-Félix 2010; Cantrell et al. 2011). They obtain nutrients in these diverse habitats by their highly variable structural and morphological forms, ranging from the unicellular yeast form to multicellular filamentous forms and a multitude of elaborate fruiting bodies. Among the multicellular fungi, there is a fascinating group that obtains their nutrients through predation and they include representatives from at least three phyla, Zygomycetes, Ascomycetes, and Basidiomycetes (Li et al. 2000). The predatory ability has originated independently multiple times in fungi (James et al. 2006). Species in genera, such as *Arthrobotrys* and *Dactylella* can lure, then trap, snare, or grip nematodes and other small invertebrate animals in soils and in wood (Barron 1977). This capacity to obtain nutrients from animals could increase their nitrogen intake and have likely contributed to their adaptation among diverse ecological niches (Schmidt et al. 2007).

Predaceous fungi are a special ecological group and include nematode-trapping fungi, endoparasitic fungi, opportunistic pathogenic fungi, and toxic fungi. Taxonomically, nematode-trapping hyphomycetes (teleomorph: Orbiliaceae) contain the largest group of predaceous fungi. These fungi are predominantly soil-living organisms that have the ability to form infection structures, like adhesive hyphae, traps, and knobs, nonconstricting rings and constricting rings to capture nematodes. The taxonomy and diversity of nematode-trapping fungi have been primarily studied using traditional morphological methods and rRNA sequences. These studies identified that the type of nematode-trapping devices was a highly reliable indicator about the evolutionary relationships among these species (Ahren et al. 1998; Li et al. 2005; Yang et al. 2007, 2012).

Nematodes are among the most diverse and abundant groups of invertebrates and they have been found in the soil (Yeates 1979), sandy biological crust (Pen-Mouratov et al. 2011), lead/zinc mines (Shao et al. 2008), saline and freshwater lakes (Andrássy and Gibson 2007), and so on. The distribution of many soil nematode taxa is strongly influenced by factors, such as soil texture, soil temperature, and broad vegetation types. In soil, nematode individuals are found in the millions per m^2^, and they exhibit a variety of feeding types and survival strategies (Yeates and Bongers 1999). Many nematode species are parasites on plants or animals, some of them have become adapted to feeding on a range of plants found in their native habitat(s). Partly due to their limited dispersal abilities, relatively few species have been found to be obligate parasites with a narrow host range (Boag and Yeates 1998). However, they can cause huge losses in agricultural economy every year (Abad et al. 2008). Their agricultural significances have resulted in an increasing interest among scientists to use nematode-trapping fungi as biological control agents to control these parasitic nematodes (Kumar and Singh 2006; Waller 2006).

*Arthrobotrys oligospora* Fres. is the most commonly isolated and by far the most abundant nematode-trapping fungus in natural environments (Duddington 1954; Persson et al. 1996; Jaffee 2004; Farrell et al. 2006; Wachira et al. 2009). Recently, several studies indicated that *A. oligospora* had a strong ability to trap nematodes in a variety of environments, such as soil (Jaffee 2004), feces (Su et al. 2007), fresh water (Hao et al. 2005), and mangrove habitats (Swe et al. 2009), as well as heavy metal-contaminated mines (Mo et al. 2006). Its broad distribution and high abundance in nature have made *A. oligospora*, a potential biocontrol agent against nematodes in agriculture and forestry. However, to make the biocontrol effective, a comprehensive understanding of the interacting partners in the agricultural and forestry ecosystems are required. In both agricultural fields and forests, the plants, nematodes, and the nematode-trapping fungi constitute a tight tripartite system with the plants providing food to nematodes, the nematodes serving as nitrogen sources to nematode-trapping fungi, and the nematode-trapping fungus *A. oligospora* living as an endophyte in plants. For example, *A. oligospora* have been found to grow inter- and intracellularly in barley and tomato roots, but never penetrate the vascular tissues of plant roots (Lopez-Llorca et al. 2006). In addition, it also induced plant defense reactions against fungal pathogens (Bordallo et al. 2002). A similar type of interaction has also been found between animal-parasite nematodes and *A. oligospora*. Jaffee and Strong (2005) suggested that the community of bush lupines, ghost moths, isopods, and an insect-parasitic nematode all contributed to the abundance of *A. oligospora* at Bodega Marine Reserve. These existing natural interactions suggest that biocontrol agents could be applied to control nematodes in a sustainable manner. Indeed, the biocontrol method was found to be better at balancing the population diversity of Bacterial- or fungi- feeding nematodes and plant parasitic nematodes than chemical methods with urea and pesticide (Zhang et al. 2002).

However, current biological control applications using these fungi have shown relatively limited success in agricultural fields and forests. There are several potential contributing factors, including competition with other soil fungi, antifungal compounds release by other microbes, and/or disturbance of the naturally existing tripartite food chain involving plants, nematodes, and the applied fungi. While and the detailed mechanisms remain to be explored, our limited understanding of the ecology and population biology of these fungi in their natural environments could be a significant factor. We believe an effective application of *A. oligospora* or other fungi for biological control requires a thorough understanding of their population structure and their modes of reproduction. For example, an understanding of their spatial and temporal patterns of genetic variation could help determine whether different strains need to be applied at different geographic areas and/or ecological niches. The objective of this study is to analyze the genetic variation among geographic and ecological populations of a representative nematode-trapping fungus, *A. oligospora*.

Several molecular markers have been used to study the diversity and evolution of nematode-trapping fungi. Restriction fragment length polymorphism (RFLP) and DNA sequencing of the internal transcribed spacer region of the ribosomal RNA gene (ITS) region have found genetic differentiation among strains within species in the genera *Arthrobotrys* and *Monacrosporium*, revealing cryptic species among morphologically similar isolates (Persson et al. 1996; Ahrén et al. 2004; Meyer et al. 2005). Intraspecific variations derived from RFLP data and polymorphisms in serine protease genes were also observed among ecotypes of the nematophagous fungus *Pochonia chlamydospora* based on host preferences (Segers et al. 1999; Morton et al. 2003). However, the study on phylogeography among 22 isolates of a nematode-trapping fungus *Duddingtonia flagrans* from diverse terrestrial habitats showed little geographic or ecological pattern (Ahrén et al. 2004).

In this study, we used molecular markers based on single nucleotide polymorphisms (SNPs) to analyze the ecology and population biology of *A. oligospora*. SNPs refer to polymorphisms generated by nucleotide substitutions or the insertion and deletion of single nucleotides between homologous DNA sequences. SNPs have become popular molecular markers for studying a variety of biological issues. So far, most such studies have focused on model organisms, and relatively little is known about the SNPs in nonmodel organisms including many fungi (Xu et al. 2007). In this research, SNPs were analyzed to examine the following issues: (1) the level and partitioning of genetic variation within the Chinese *A. oligospora* occupying different geographic and ecological habitats; (2) Genetic structure and modes of reproduction in populations of this species. We discuss the implications of SNPs and polymerase chain reaction PCR-RFLP markers in the inference of the population structure and ecological patterns of *A. oligospora*, and the conservation and biocontrol application of this species in nature.

## Materials and Methods

### Strains

To compare the level and partitioning of genetic variation within the Chinese *A. oligospora* occupying different ecological niches, we screened four types of environments in southwestern China: (1) a polluted aquatic environment (Dianchi lake in Kunming city, Yunnan province), (2) a pristine aquatic environment (Jiuzhaigou Nature Reserve in A'ba county, Sichuan Province), (3) a high salt containing environment (a salty mine in Heijing county in Yunnan province), and (4) a heavy metal polluted environment (lead-zinc mine in Gejiu city in Yunnan province). Soil samples from forests adjacent to these four special environments were also collected simultaneously, in order to detect the relative contributions of ecological niche and geographic distance to the genetic diversity of *A. oligospora*. Samples from other two geographic sites from Xinjiang province, Kanas Nature Reserve and Turfan, were also obtained for analyses. In short, while two geographic populations in Xinjiang province contained one ecological population each (both were from soil samples), the other four geographic populations from Sichuan province and Yunnan province contained two ecological populations each, with one from natural forest soils and the other from aquatic environments or mines. The detailed information of sampling sites and habitats are given in Table 1. The collecting methods of soil and aquatic samples followed those of Li et al. (2000) and Hao et al. (2005). The aquatic samples were obtained from the surface of the sediments.

**Table 1:** Geographic distribution and genotype diversity of the *Arthrobotrys oligospora* strains we analyzed in this study

Geographic population/(Province)	Collecting site	Sample size	Habitat	Latitude	Longitude	Altitude (m)	Multilocus genotype (no. of isolates in each genotype)	Multilocus genotype diversity	Percentage of Polymorphic Loci (*P*%)
Dianchi (Yunnan)	Dianchi Lake	29	Polluted water	25.00	102.41	1895	4 (1);15 (1);19 (1);23 (1);33 (1);34 (1);38 (1);60 (1);61 (1);62 (1);64 (1);67 (1);69 (1);70 (1);73 (1);83 (1);88 (1);90 (1);95 (1);97 (1);102 (1);104 (1);105 (1);108 (1);117 (1);120 (1);128 (1);130 (1);52 (1)	0.447	100
	Xishan Mountain	21	Forest soil	25.00	102.41	1895	8 (1);39 (1);40 (1);65 (1);75 (1);80 (1);81 (8);85 (7)	0.213	50
Heijing (Yunnan)	Ancient salt mine	28	high salt containing soil (100 g/liter^−1^ total dissolved salts)	25.21	101.44	1700	2 (2);3 (1);17 (1);43 (1);44 (1);47 (1);48 (1);54 (1);56 (1);77 (1);79 (1);87 (1);91 (1);98 (1);111 (1);112 (1);113 (1);114 (1);116 (3);121 (1);127 (1);129 (1);131 (1);138 (1);141 (1)	0.319	91.67
	Feilai Mountain	20	Forest soil (3.6 g/liter^−1^ total dissolved salts)	25.21	101.44	1700	42 (1);57 (1);58 (1);59 (3);103 (1);107 (2);109 (1);110 (1);122 (1);123 (2);124 (1);125 (1);126 (1);133 (1);134 (1);142 (1)	0.301	75
Gejiu (Yunnan)	Laochang mining area	30	Heavy metal polluted soil (Pb 16932 mg/kg; Zn 15579 mg/kg; Cd 152 mg/kg)	23.21	103.09	1688	1 (1);6 (1);7 (1);9 (1);11 (1);12 (1);14 (1);16 (1);18 (2);20 (1);21 (1);23 (1);27 (1);28 (1);44 (1);53 (1);55 (1);66 (1);71 (1);72 (1);76 (1);77 (1);78 (1);86 (1);90 (1);118 (1); 137 (1); 139 (1); 140 (1)	0.336	83.33
	Laochang Mountain	21	Forest soil (Pb 810 mg/kg; Zn 520 mg/kg; Cd <50 mg/kg)	23.21	103.09	1688	10 (1);13 (1);41 (1);63 (1);74 (1);81 (7);82 (1);85 (1);99 (1);100 (1);101 (1);132 (1);135 (1);136 (1); 143 (1)	0.332	75
Jiuzhaigou Nature Reserve (Sichuan)	Nuorilang	29	Forest soil	32.55	103.16	2600	32 (2);35 (1);36 (1);45 (1); 49 (1); 68 (1); 84 (1);90 (1);92 (1); 93 (2); 94 (1);95 (1); 96 (4); 106 (1);116 (4);115 (3); 119 (3)	0.336	91.67
	Panda Lake	16	Pristine aquatic area	32.55	103.16	2600	36 (5);37 (3);46 (3);50 (1);51 (1);96 (1);115 (1);116 (1)	0.198	50
Turfan (Xinjiang)	Desert Botanical Garden	5	Desert soil	48.2	87.34	−87	24 (3); 26 (1);30 (1)	0.074	16.67
Kanas (Xinjiang)	Kanas Forest	29	Forest soil	48.81	87.04	1370	5 (2);22 (4);24 (16);25 (1);29 (1);30 (1);31 (2); 89 (2)	0.096	33.33

Strains of *A. oligospora* were obtained using the standard “sprinkle plate” method in which a small quantity of substrate was placed on agar and entomopathogenic “bait” nematodes were added to selectively enhance the growth of nematode-trapping fungi (Duddington 1951; Gray 1983; Li et al. 2000). Taxonomic characters were examined from cultures on CMA 7–14 days after inoculation. The sizes of conidia and conidiophores were obtained using an Olympus BX51 microscope (Olympus Corporation, Tokyo, Japan).

### DNA extraction, molecular identification and genomic library construction

Genomic DNA was extracted from the mycelia collected from single-spore cultures growing on cellophane membrane on PDA medium according to the modified CTAB method (Yu et al. 2007). ITS sequences of 31 randomly selected strains representing all ecological niches were obtained according to the method described by Zhang et al. (2011) to further confirm the morphological identification of these *A. oligospora* isolates.

A random shotgun genomic library was constructed using genomic DNA from strain DC2 according to the methods described previously (Xu et al. 2007). The cloned fragments in the range 0.5–1.0 kb were amplified using vector primers and the PCR products were confirmed and then sequenced.

### SNPs identification

From the above obtained sequences, PCR primers were designed after the flanking vector sequences were firstly trimmed. These primers were used to directly amplify and sequence the DNA from two strains DC2 (as positive control) and WCC3 (that strain being geographically and ecologically different from DC2). The obtained sequences from the two strains were compared with identify SNPs.

### Genotype

A total of 12 polymorphic fragment-restriction enzyme combinations based on the above identified SNPs were chosen to genotype the entire 228 strains. These polymorphic sites are distributed on eight random DNA fragments. They are *Hin*dIII at fragment #2 and #47, *Nae*I, *Eco*RI and *Pvu*II at fragment #4, *Eco*RI at #15, *Dra*I, and *Xho*I at fragment #26, *Apa*lI at fragment #55, *Apa*I at fragment #57, *Bpu*1102I, and *Nru*I at fragment #76. For strain genotyping, the DNA fragments that contained SNPs detectable by restriction enzyme digests were first amplified using PCR. After confirmation of the PCR products using agarose gel electrophoresis, the fragments were digested using the specific restriction enzymes. PCR products and restriction digests were electrophoresed in 1.0% agarose in 1× TBE, the genotypic data were scored by the electrophoresis bands viewed under ultraviolet transillummination (Xu et al. 2008).

### Data analysis

For each PCR-fragment, the RFLP pattern were scored based on the presence/absence of specific DNA fragments after restriction enzyme digests and gel electrophoresis. Each unique DNA fragment pattern for each PCR fragment-restriction enzyme was assumed to represent a specific allele at a single genetic locus. Each combination of alleles at the 12 loci defined a multilocus genotype. Isolates with the same multilocus genotype were considered being part of a clone. All analyses were performed using clone-corrected data set, that is, each clone was represented by exactly one entry in the data sets.

### Population structure

Analysis of potential population subdivision as a function of habitat and geographical origin was carried out to test whether the analyzed populations were genetically different from each other. We first used principal components analysis (PCA) to produce a two-dimensional visual summary of the observed genetic variation (Patterson et al. 2006). The Bayesian methods were then used to study the affiliation of individual isolates from sampling locations to specific clusters and test for admixture (Pritchard et al. 2000; Beaumont and Rannala 2004). For the LOCPRIOR model with admixture and correlated allele frequencies, the program STRUCTURE version 2.3.3 (Hubisz et al. 2009) was used to explore the number of genetic clusters (*K*) occurring in the sample. A total of 30 replicates were performed of each simulation for *K* = 1–11, with a burnin of 10,000, and Markov Chain Monte Carlo (MCMC) of 100,000 iterations for the best fixed value of *K*. Finally, based on results indicated in the above mentioned analyses, the contributions of different levels of separation (i.e., regional geographic populations and ecological populations) to the overall genotypic diversity were assessed through the analysis of molecular variance (AMOVA). In this analysis, each regional population represented a geographic population, whereas the two ecological samples from each of the four regions represented subpopulations within each geographic population (Table 1).

### Genetic variance

The clone-corrected multilocus genotypic data were directly imported into GenAlEx6 version 6.3 (Peakall and Smouse 2006) to calculate genotype diversity, and the pairwise population genetic distances (Nei's genetic distance). The geographic distances and altitudinal differences between local populations were calculated between each pairs of populations based on their geographic coordinates at each location. The quantitative relationship between genetic difference and geographical parameters (geographic distance) were assessed using the Mantel test (Dietz 1983).

Genetic relationships among geographic populations of *A. oligospora* was further estimating by theta (Weir 1996), using MultiLocus 1.3 (Agapow and Burt 2001). The null hypothesis of this test was no genetic differentiation among populations as defined by geographical origins. Statistical significance for this test was derived by comparing the observed dataset to 1000 randomized datasets generated assuming no genetic differentiation. During randomization, any linkage disequilibria present in the observed data were removed. The statistic θ ranges from 0 (for no population differentiation due to frequent gene flow) to 1 (total isolation of the populations from each other).

### Clonality and recombination

The anamorphic (asexual stage) *A. oligospora* has a corresponding teleomorph (sexual stage) *Orbilia auricolor* (Pfister 1995). However, the sexual stage has been difficult to observe in laboratory cultures or in nature. To examine whether there is evidence of recombination in individual ecological populations and the role of sexual recombination in natural populations of this species, the following three complementary tests were conducted. First, we calculated the index of association (I_A_) and rBarD. The null hypothesis for I_A_ is that there is random association (recombination, linkage equilibrium) among alleles at different loci. A *P*-value of <0.05 would indicate that the null hypothesis should be rejected, and statistical significance was derived by comparing the observed dataset to 1000 simulated datasets generated assuming random recombination. The pairwise linkage disequilibria were also measured to assess the role of recombination to the genetic diversity in each population. We standardized the I_A_ value by the number of loci with the rBarD algorithm adjusting for the numbers of loci, because the value of the traditional I_A_ can be influenced by the number of loci analyzed (typically the higher the number of loci, the higher the I_A_ value). The adjustment helps facilitate better comparison between populations and studies.

In the second test, the proportion of pairwise loci that were phylogenetically incompatible (PI) was calculated. Phylogenetic incompatibility is an indicator of recombination at the population level. In contrast, the lack of phylogenetic incompatibility implies asexual reproduction. In the simplest case, in a haploid species (like *A. oligospora*), a phylogenetic incompatibility occurs between two loci with two alleles each when all four possible genotypes are found in the population. The incompatibility ratio (IR), where IR = (number of incompatible pairs of sites in the test dataset)/(number of incompatible pairs of sites in a randomly shuffled dataset), can be used as a test for inferences of statistical significance (Agapow and Burt 2001). Both the phylogenetic incompatibility and the RbarD tests were conducted using MultiLocus 1.3 (Agapow and Burt 2001).

In the third test, Parsimony Tree Length Permutation test (PTLPT), commonly used phylogenetic methods developed to test for signals of phylogenetic tree congruence (Archie 1989), was adopted to test for reproductive mode (Burt et al. 1996). The PTLPT uses the permutation test in PAUP* (version 4.0b10) (Swofford 2002) to calculate the length of phylogenetic trees by treating the isolates as taxa and the alleles at each locus as phylogenetic characters with two character states. The analyses involved comparing the values for the observed data set with the values for 1000 artificially recombining data sets. Artificially recombining data sets were constructed by randomly shuffling the alleles for each locus between members of the population while keeping the proportions of alleles at each locus constant. The inability to distinguish between the observed data set and the artificially recombined data sets supports the null hypothesis of sexual recombination, whereas a significant difference between the observed and permuted data sets supports clonality.

## Results

### Sampling and identification of SNPs

From 1600 environmental soil/water sediment samples, a total of 228 *A. oligospora* strains were isolated. These strains were grouped into six geographic populations containing 10 subpopulations total (Table 1). The BLAST searches in the GenBank showed that sequenced ITS fragments of 31 randomly selected strains showed lower than 3% dissimilarity to the published ITS sequences of *A. oligospora*, consistent with morphological identifications. PCR primers of 21 cloned fragments were designed to amplify 9694 bp sequences from the genomic DNA of DC2 (Table 2). The genomic DNA of another natural strain WCC3 isolated from the forest soil of Jiuzhaigou Nature Reserve, Sichuan province, was also used as template to amplify the 21 fragments, from which 9761 bp were obtained. In total, 268 SNPs were identified between these two strains, representing a SNP frequency of 2.76% per nucleotide site. The comparative analysis of these two strains revealed that all 21 fragments contained SNPs, the SNPs frequency ranged from 0.24% to 9.51% per fragment. The 268 SNPs included 200 transitions and 54 transversions, and 14 indels.

**Table 2:** Primers used to screen for single nucleotide polymorphisms and haplotypes within and between strains of *Arthrobotrys oligospora*

Fragment name	Primer sequence (5′→3′)	Expected size (bp)
2#	Forward: ATATGTCGCCCGCGTTCTAGAGCAA	375
	Reverse: TCAAGTCCCTGTCTTTCCTCCTCAC	
4#	Forward: TTCAAATACTACAGACCCACTTCG	643
	Reverse: AACTTTCCACGCCCTCACT	
6#	Forward: TCGACGGACAGAAGGTTGCA	373
	Reverse: GCTGGCGTTATCTTGGGCAT	
10#	Forward: GCCGATAGAGCAGAGCAAGT	447
	Reverse: CGAAGTGATACCCGCAGGT	
15#	Forward: ATCCACCCGACAATCCCAT	688
	Reverse: CGGCACAGAGAAGTCAAAGC	
26#	Forward: CTGCGATAGGTGAGCGACTC	536
	Reverse: CCTGCCAACATCCGATTCG′	
30#	Forward: CCTTGCCACAAACCTACCGTC	412
	Reverse: CCTCCCGCATCTTCTCTCAATAC	
34#	Forward: GGTGTGACTCGGAAAGATGGTAAG	528
	Reverse: ATGGCTTGCTCGCTCGGTAG	
38#	Forward: TCTACTGCGACCTCCAAATGC	436
	Reverse: TTGATACACCTTCACTGCTACATCC	
39#	Forward: ATCTTCGTAAGCCGTCGCA	430
	Reverse: GAACATTCCTCGGGTAGCCT	
42#	Forward: ATCCTTCTTCCAGCCATCAATAC	479
	Reverse: CGGTTTGGGACTTTGCGGA	
47#	Forward: TGTTCAGTAATCCGCCTCATAG	555
	Reverse: TTTCCCGAGTCTCAATCCGA	
48#	Forward: GAGGCAGATTGATGATGAGAAGG	621
	Reverse: ATGGAGGAAAGCGGATGCGA	
55#	Forward: CTCCCTTCCCTTCCAATCCA	284
	Reverse: TACCATCATCAGCCGTCCA	
57#	Forward: GGGCAGAAACCGTAAGTAACT	483
	Reverse: TCTCCCATCGCTTGTCCTT	
75#	Forward: ATCTTTGGCTCCTTCGTGGC	391
	Reverse: TCGTGCTCGGAATCCTCAA	
76#	Forward: TGGAGAGCACTAACACGATGT	400
	Reverse: AGGGTGACCAAGAGAGTAACA	
82#	Forward: TCGCCCAAGAAGATAAATCGC	302
	Reverse: TGAGGACTGATGAGAGTGAAGA	
85#	Forward: TACCCGATTCCCGACACCTA	461
	Reverse: ATCCTCCGTCTCTTCGCTTC	
86#	Forward: TTGCTCGGGTATCGTCGTCA	471
	Reverse: GATGTTTACTCTCCACGGCG	
87#	Forward: TGTGAATGTGAGTGTGAACGG	369
	Reverse: TGCCAACAGGAGTCTAACAAC	

**Figure 1:**
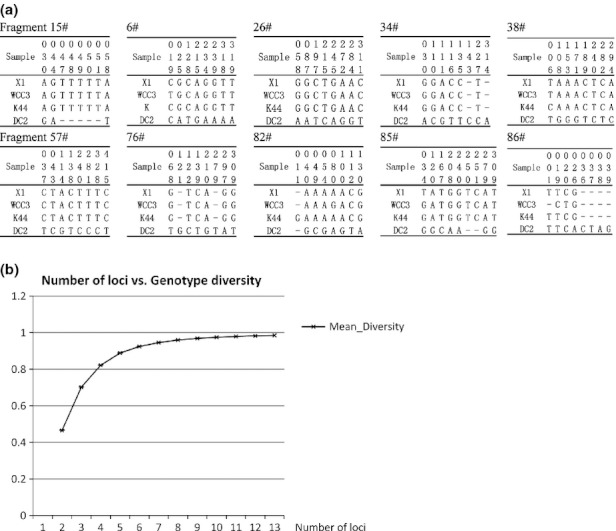
(a) Parts of the sequence aligments of all four strains (DC2, WCC3, K44, and X1). For each panel, the top row indicates the random clone fragment. The second row contains three digits, indicating the nucleotide position of each single nucleotide polymorphism in the sequenced fragment, reading from top to bottom. (b) Permutation analysis showing the relationship between the number of assayed lici and genotype diversity in the total samples of *Arthrobotrys oligospora* strains from China.

The 21 primer pairs were also used to amplify homologous sequences from two additional isolates K44 and X1 obtained from forest soils of Kanas Nature Reserve and Turfan, respectively. Sequence alignments of all four strains (DC2, WCC3, K44, and X1) showed that strains WCC3, K44, and X1 were highly similar to each other. whereas strain DC2 was different from those three in several fragments (Fig. 1a). These four isolates were geographically distant from each other, but strain DC2 was isolated from a lake polluted by industrial and household wastes, whereas the other were all from pristine forest soils.

### Genetic diversity of *A. oligospora* across different geographic regions and ecological niches

To analyze the genetic diversity of *A. oligospora* across geographic regions and ecological niches in western China, 12 polymorphic restriction sites distributed on eight of the 21 sequenced fragments that could be easily amplified in all populations were used to genotype all 228 *A. oligospora* strains. Our analyses identified a total of 143 genotypes. The clone-corrected dataset which was used for the following analyses included 143 individuals.

The overall genotypic diversity for the whole sample was 0.265, and there was a range of variation in genotypic diversity among the 10 subpopulations, from a low of 0.074 in Turfan population to a high of 0.447 in Dianchi population. The genotypic diversity and number of repetition of these genotypes were presented for each sub-population in Table 1. When comparing genotypic diversities between pairs of ecological subpopulations, we found that in three of the four regions, those from aquatic and/or polluted environments were always higher than pristine soil populations (Table 1).

**Figure 2:**
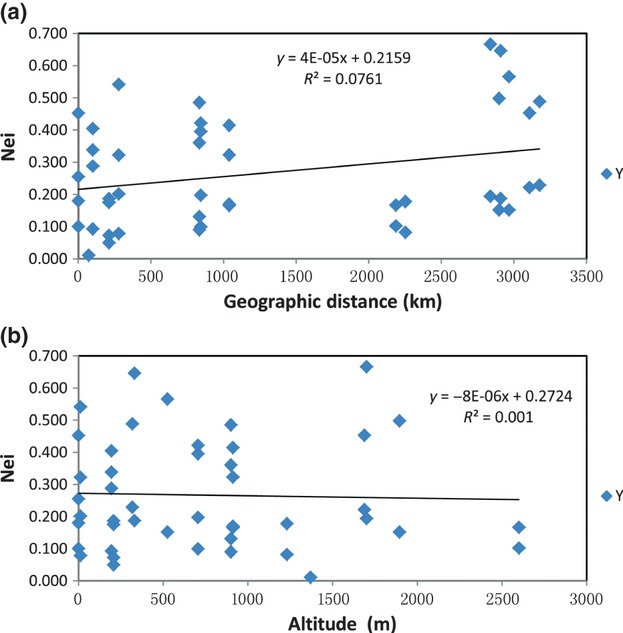
Results from two Mantel tests between genetic differences and geographic distances among populations. (a) A Mantel test between Nei's genetic distance and the two-dimensional geographical distances (based on longitudinal and latitudinal coordinates) among populations. (b) A Mantel test between Nei's genetic distance and altitudinal differences between populations.

DNA sequence divergence among populations was estimated using pairwise Nei's genetic distance. The lowest value of 0.011 was found between Kanas and Turfan populations from Xinjiang Province. The highest distance was 0.666, between forest soil populations from Heijing and Turfan populations from Xinjiang Province. The Mantel test indicated a positive correlation between genetic and geographical distances, with a correlation coefficient of 0.276. However, the correlation was statistically insignificant (Fig. 2a; *P* = 0.18). No correlation was found between altitudinal differences and population genetic distances (Fig. 2b; correlation coefficient = −0.031, *P* = 0.52).

### Genetic structure

**Figure 3:**
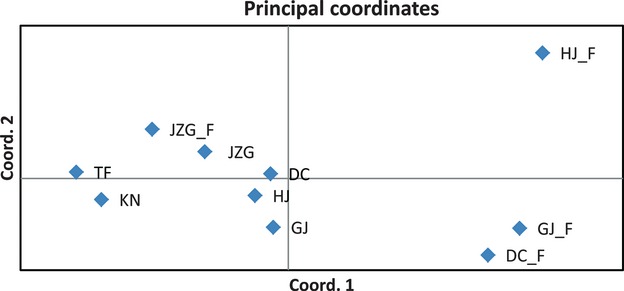
Principal components analysis of *Arthrobotrys oligospora* strains from China. Note: DC, Dianchi polluted lake; HJ, Heijing salt mine, JZG, Pristine aquatic environments from Jiuzhaigou Nature Reserve; GJ, Gejiu heavy metal mine; KN, Kanas Forest, TF, Turfan Desert Botanical Garden, Populations originated from soil samples from the forest, which is adjacent to the four special environments were marked with “_F.”

Using PCA, populations from Dianchi lake (polluted aquatic environment), Gejiu City (lead-zinc mine), and Heijing county (high salt containing environment) grouped together and were distinct from populations from forests adjacent to these three special environments. However, the two sub-population from Jiuzhaigou Nature Reserve (pristine aquatic environment and forest soil) were grouped together (Fig. 3). The direction of the PC1 axis and its relative strength may reflect a special role for this ecological axis in the genetic structure of *A. oligospora*. PC1 accounts for approximately twice the amount of variation as PC2 (41.89% vs. 23.09%).

**Figure 4:**
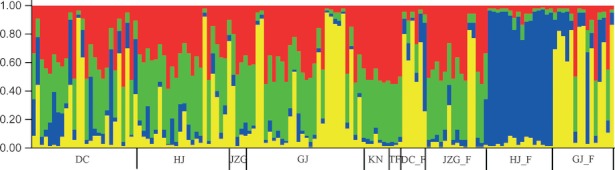
Bar plot of individuals' ancestry as inferred from Bayesian clustering with STRUCTURE for clone-corrected genotypic data. Number of groups is *K* = 4. Individual isolates are represented by one line and populations were marked on the bottom. The abbreviations of population name are same as Fig. 3.

Bayesian clustering using STRUCTURE on the 143 genotypes did not reveal any clusters based on their geographic origins, consistent with the absence of a clear geographic structure among the *A. oligospora* strains (Fig. 4). The number of clusters *K* = 4 was chosen because standard deviation of posterior probability is the lowest for that *K*. Standard deviations increase with higher *K* values, suggesting weaker support for higher *K*. However, a general picture of admixture emerged in our analysis and some isolates from each subpopulation showed clustering different from other isolates of the same subpopulation. Figure 4 describes the percentages of individuals of each sampled subpopulation that belongs to each of the derived clusters for *K* = 4, which captures the major structure in this data set. Our results also suggest that the ancestry of several genotypes in the 10 subpopulations could be traced to more than one cluster. Several genotypes from one subpopulation shared alleles with other subpopulations, indicating either that these 10 subpopulations likely shared a recent common ancestry and/or there was recent migration and genetic recombination among these subpopulations.

Contrarily to the lack of genotype clustering based on geographic origins, ecological factors showed an obvious effect on the clustering pattern. When detecting clustering patterns between subpopulations in the same geographic region, but occupying different ecological niches, we found great differences between population pairs that had samples from polluted aquatic, high salt containing, and heavy metal polluted environments (e.g., DC vs. DC_F, HJ vs. HJ_F, and GJ vs. GJ_F). The most evident differences in cluster pattern were found between high salt containing population and its counterpart from forest soil (HJ vs. HJ_F). However, membership assignments to the inferred genetic clusters revealed similar patterns between populations from pristine aquatic environment and its corresponding forest soils (JZG vs. JZG_F), and between populations from Kanas Nature Reserve and Turfan (KN vs. TF), even though they are geographically separated by over 300 km.

The 10 subpopulations showed significant genetic differentiations, with a θ value of 0.3293, significantly larger than the randomized datasets (*P* < 0.001). The analysis of molecular variance (AMOVA) revealed that 24% of the total genetic variation was due to ecological niche separations, and geographic distributions showed no contribution to the total genetic variation. The majority of the genetic variance (76%) was found within individual subpopulations (Table 3).

**Table 3:** Summary results of analysis of molecular variance (AMOVA) within and among populations of *Arthrobotrys oligospora* from China

Source	df	SS	MS	Est.Var.	%var	AMOVA Statistics	Value	*P*
Among Regions	5	47.325	9.465	0.000	0	PhiRT	−0.053	1.000
Among Populations	4	51.975	12.994	0.770	24	PhiPR	0.239	0.010
Within Populations	133	326.588	2.456	2.456	76	PhiPT	0.199	0.010
Total	142	425.888		3.226	100			

### *A. oligospora* reproductive mode

The multilocus linkage disequilibrium test rBarD and phylogenetic incompatibility tests were conducted for (1) the total sample, (2) samples from each geographic and ecological populations (Table 4). Due to its small sample size, the population from Turfan was excluded from these analyses. Our analyses identified that rBarD values and the lengths of the most parsimony trees made from the observed data set for the total sample, Jiuzhaigou_forest, Heijing_salt mine, Gejiu_forest, and Gejiu_lead-zinc mine were all significantly different from the null hypothesis of random recombination. The results suggest a predominantly clonal population structure for these sample sets. However, the phylogenetic incompatibility test identified evidence of recombination in all sample sets, a result indicative of limited recombination. Variable levels of recombination were found within different subpopulations in the same geographic regions. Interestingly, while the subpopulation from Jiuzhaigou forest had higher levels of phylogenetic incompability than its corresponding aquatic subpopulation, in the three other geographic areas, subpopulations from stressful environments had higher levels of phylogenetic incompability than the corresponding forest soil samples in Dianchi, Heijing, and Gejiu. The same results were found based on the percentages of loci pairs in linkage disequilibria in the pairwise comparisons between different DNA fragments, with those from stressful environments showing higher ratio of phylogenetic incompatibility than the corresponding forest soil samples in Dianchi, Heijing, and Gejiu.

**Table 4:** Multilocus linkage disequilibrium analyses form samples of *Arthrobotrys oligospora* from China

				Percentages of linkage disequilibria^1^ (%)	Tree length of PTLPT test		
					
Sample set	Sample size	rBarD (*P* value)	PrC (*P* value)		Observed	Randomized dataset	*P* value
Total sample	143	0.030 (<0.001)	0 (1.0)	14.7	206	242–273	<0.001
Dianchi Lake	26	0.014 (0.074)	0.015 (0.829)	0	57	51–65	0.228
Dianchi_Forest	6	0.037 (0.843)	0.909 (0.248)	9.8	13	11–15	0.449
Jiuzhaigou_aquatic	4	0.045 (0.631)	N/A	22.9	6	6	1
Jiuzhaigou_forest	15	0.105 (<0.001)	0.606 (0.041)	8.2	21	21–30	0.003
Heijing_salt mine	23	0.064 (<0.001)	0.318 (0.115)	3.2	47	42–54	0.254
Heijing_forest	16	0.012 (0.524)	0.636 (0.075)	19.7	23	18–27	0.628
Gejiu_lead-zinc mine	29	0.062 (0.003)	0.348 (0.003)	18	45	50–63	<0.001
Gejiu_forest	15	0.188 (0.004)	0.530 (0.080)	36.1	23	21–31	0.031
Kanas	6	N/A	N/A	18	7	7	1

PTLPT, Parsimony Tree Length Permutation test.

1 Percentages of linkage disequilibria (*P* < 0.05) in the pairwise comparisons between different fragments (%)

## Discussion

### SNP genotyping assays

Because of its relatively low mutation rate and ubiquitous nature, SNP markers have provided insights into the genetics and population history of many organisms, including fungi (Brumfield et al. 2003). For example, the intraspecific SNPs have been characterized for many fungal species, such as *Candida albicans* (Forche et al. 2004), *Fusarium* spp. (Kristensen et al. 2007), *Cercospora beticola* (Groenewald et al. 2007), *Coccidioides immitis* (Fisher et al. 1999), *Tricholoma matsutake* (Xu et al. 2007), and the entomopathogenic fungus *Pandora neoaphidis* (Fournier et al. 2010).

Here, we reported the SNP distributions and frequencies for the first time for a nematode-trapping fungal species. We identified 268 SNPs distributed among 21 cloned fragments, with an average SNP frequency of 2.76% per nucleotide site. The frequency is comparable to those observed in several other fungal species. For example, there is a SNP frequency of 2.05% per nucleotide in the model yeast *Saccharomyces cerevisiae* (Ayoub et al. 2006), 3.4% in *Candida parapsilosis* (Fundyga et al. 2004) and 1.707% in the ectomycorrhizal mushroom *T. matsutake* (Xu et al. 2007). However, the whole SNPs in the *A. oligospora* will likely be much higher than 2.76%. This is because our current estimate was primarily based on comparing two strains (DC2 and WCC3) collected from terrestrial and aquatic environments, respectively. Sequence alignments with two additional isolates (K44 and X1) from forest soils of Kanas Nature Reserve and Turfan, identified more, although somewhat limited number of additional SNPs. The comparatively large difference between strain DC2 (from a polluted lake) and the other three strains from pristine forest soils suggest that pollution and environmental stress might have contributed to ecological divergence for populations in such environments.

On the other hand, the nucleotide substitution pattern in our fungus is quite different from several other organisms. The transition to transversion ratio in *A. oligospora* was approximately 5, which is quite similar to that of the recently reported mushroom species *T. matsutake* (Xu et al. 2007), but is higher than that of *C. albicans*, *Cryptococcus neoformans*, and *Candida guilliermondii* (all about three in each species) (Xu et al. 2000; Forche et al. 2004; Lan and Xu 2006). The lowest ratio found so far was ∼1.4:1 in *C. parapsilosis* (Fundyga et al. 2004) and 1.9 in some species of the class Pyrenomycetes (Ascomycotina) (Berbee and Taylor 1992). It is well known that the rate of transitional substitutions differs from the rate of transversional subtitutions, with transitions generally occurring more frequently than transversions. This difference is often referred to as transition bias, and estimation of the extent of transition bias may be of interest, as it may vary among organisms and among genes within the same organisms. The knowledge of this quantity may aid in our understanding of the patterns of molecular evolution. The mechanism(s) responsible for the transition bias in the nucleotide substitution patterns among *A. oligospora* and their potential evolutionary significance are worthy of further investigation, especially on its transposable element content and its location in the genome of this fungus.

Some of the above mentioned SNPs were further distinguished using simple PCR followed by digestions using specific restriction enzymes to generate RFLP. We genotyped 228 strains from 10 subpopulations, including two from two sites in Xinjiang province and two each from four other geographic regions. At each of the four regions, one sample was from the pristine forest soil environment and the other was from an aquatic and/or polluted environment. The results from randomizations conducted in Multilocus 1.3 showed a level of saturation for identifying unique genotypes using the eight DNA fragments and 12 RFLP loci, suggesting that the total 12 loci analyzed here were sufficient to obtain robust genotypes of the strains (Fig. 1b). The SNP assay provided an efficient tool for investigating population structures and diversity of *A. oligospora*. Furthermore, these markers may constitute a powerful approach for monitoring the spatial and temporal dynamics of strains of *A. oligospora* used for nematode biocontrol applications in agricultural fields and forests.

### Contributions of geographic and ecological niche separation to genetic differentiation

Our analyses identified that ecological niches exerted more effect on genetic diversity and differentiation than geographic separation among subpopulations of *A. oligospora* in China. Nematodes are ubiquitously distributed in nature and we expect that they exist in our tested stressful environments. Although some nematode-trapping fungi are obligate parasites of nematodes (Hallmann et al. 2009), most (including *A. oligospora)* are facultative parasites. Nematodes in these environments could activate the parasitic behavior from the saprophagous habitats (Pathak et al. 2012). If nematodes in the stressful environments were unique and played an important role in the nematode-fungal interaction, these nematodes could be a significant selective forms in determining genotype distributions in these environments. In addition, the specific stress factors in the environment could also be important for determining *A. oligospora* genotypes. Evidences of genetic differentiation based on habitats have also been reported for several other microorganisms. For example, the genomic divergence of *Bacillus simplex* strains was found to significantly correlate with ecological contrasts, but not with geographic distance (Sikorski and Nevo 2005). In another study, the fungal pathogen of pine forests, *Grosmannia clavigera,* from the same host species in different geographic areas were genetically closer than those collected from different host species occurring in the same geographic region (Alamouti et al. 2011). Similar findings were also reported for other fungal pathogens, such as *Metarhizium anisopliae* (Bidochka et al. 2001), *Claviceps purpurea* (Douhan et al. 2008), *Venturia inaequalis* (Gladieux et al. 2011), fungal endophyte *Colletotrichum gloeosporioides* agg. (Gazis et al. 2011), and the arbuscular mycorrhizal fungi (Li et al. 2010).

DNA sequence variation among strains of *A. oligospora* suggested a general pattern of continuous range expansion coupled with recent local adaptation within individual lineages and sublineages (Zhang et al. 2011). Here, the divergent genetic differentiation caused by ecological niche separation confirmed the importance of local adaptation and ecological niche specialization in these environments. The lower level of differentiation among geographical populations suggests that there is long-distance dispersal and frequent gene flow due to predominant clonal reproduction between populations of *A. oligospora*.

### Ecological stress and recombination in *A. oligospora* populations

Stressful conditions are known to increase genetic polymorphism, recombination, mutation, gene conversion, and sex in biological organisms (Korol et al. 1994), in changing environments, recombination could allow more rapid adaptation (West et al. 1999). Several studies have revealed strong positive correlations of diversity and stress under conditions near the limits of growth and reproduction. Indeed, it has been suggested that increases in the mutation rate, recombination, and gene conversion may ensure higher levels of genetic diversity, providing greater potential for genetic adaptation (Nevo 2001; Kis-Papo et al. 2003). In plant fungal pathogens, a high level of recombination provides an advantage in rapidly generating many new combinations of virulence genes to counterbalance corresponding resistance genes in the host (Chen and McDonald 1996; Zhan et al. 2003; Souza et al. 2010). In arbuscular mycorrhizal fungi, recombination or recombination-like events in addition to clonality have greatly contributed to genetic diversity (Vandenkoornhuyse et al. 2001).

In our study, as expected, a predominantly asexual reproductive mode was found for the whole samples. However, we also found unambiguous evidence for recombination in populations from polluted aquatic, high salt containing, and heavy-metal polluted environments. As different genotypes may display different fitness in different environments, mutation or recombination could have slowed the loss of genetic variation due to genetic drift and local selection in these populations. The high genotype diversity in more stressful conditions likely reflected the adaptive significance of high genetic diversities in stressful environments. The high genotype diversity was partly due to the increased rate of recombination in stressful environments, as demonstrated in our tests. This result is consistent with previous observations that recombination frequencies tend to increase under stressful conditions (Hoffmann and Parsons 1991; Korol 1999). Aside from mutation and recombination, gene flow could also contribute to increased genetic diversity. This hypothesis is supported by the results from Jiuzhaigou Nature Reserve where the subpopulation from pristine forest soils was higher than those from the pristine aquatic environments. At this site, the aquatic subpopulation was likely a subset of the soil population derived from soil runoff. Indeed, the genetic assignments for strains from these two ecological niches in Jiuzhaigou were very similar to each other.

It is known that both asexual and sexual reproductive modes can confer significant advantages as well as disadvantages to fungal populations (Sun and Heitman 2011). Here, a general picture of admixture emerged in our analysis. It appears that different *A. oligospora* populations may have different reproductive strategies, and the levels of genotypic diversity were at least partly associated with the levels of recombination. How this variation in reproductive mode across *A. oligospora* populations is controlled and whether these distinct populations use either sexual and/or asexual reproductive modes to regulate gene flow, to select more rapidly adapted genotypes, and to respond to heterogeneous environments remain open questions. Extensive environmental sampling and laboratory mating assays would allow full understanding the mating behavior and differentiating the relative contribution of clonal versus sexual reproductive modes in this fungus.

Given the close relationship between ecological divergence and reproductive isolation and consequently the presumed role of ecological adaptation in speciation, an evaluation of the studied organism's ecology could also help species delimitation in nematode-trapping fungi. A general decrease in genome-wide within-population genetic variation, and an increase in between-population genetic variation should be tested using adaptive genes that are directly involved in responding to environmental changes. These genetic differences could be compared with phenotypic variation among genotypes in their physiological ability to cope with variable microenvironments. Understanding the ecological adaption of this fungus could help design sustainable and effective biocontrol application strategies. For example, because *A. oligospora* seemed to have a strong ability to increase the rates of mutation and recombination under environmental stress, it might be better to select wild strains from stressed soils as biocontrol strains. Such strains could generate recombinant genotypes by crossing with native strains and enhance their environmental adaptability and parasitizing ability. Additional field investigations are needed to evaluate the effectiveness of this strategy.

## References

[b1] Abad P, Gouzy J, Aury JM, Castagnone-Sereno P, Danchin EG, Deleury E (2008). Genome sequence of the metazoan plant-parasitic nematode *Meloidogyne incognita*. Nat. Biotechnol.

[b2] Agapow P-M, Burt AC (2001). Indices of multilocus linkage disequilibrium. Mol. Ecol. Notes.

[b3] Ahren D, Ursing BM, Tunlid A (1998). Phylogeny of nematode-trapping fungi based on 18S rDNA sequences. FEMS Microbiol. Lett.

[b4] Ahrén D, Faedo M, Rajastiekar B, Tunlid A (2004). Low genetic diversity among isolates of the nematode-trapping fungus *Duddingtonia flagrans*: evidence for recent worldwide dispersion from a single common ancestor. Mycol. Res.

[b5] Alamouti SM, Wang V, DiGuistini S, Six DL, Bohlmann J, Hamelin RC (2011). Gene genealogies reveal cryptic species and host preferences for the pine fungal pathogen *Grosmannia clavigera*. Mol. Ecol.

[b6] Andrássy I, Gibson J (2007). Nematodes from saline and freshwater lakes of the Vestfold Hills, East Antarctica, including the description of *Hypodontolaimus antarcticus* sp. n. Polar Biol.

[b7] Archie J (1989). A randomization test for phylogenetic information in systematic data. Syst. Zool.

[b8] Ayoub MJ, Legras JL, Saliba R, Gaillardin C (2006). Application of multi locus sequence typing to the analysis of the biodiversity of indigenous *Saccharomyces cerevisiae* wine yeasts from Lebanon. J. Appl. Microbiol.

[b9] Barron GL (1977). The nematode-destroying fungi. [Topics in Mycology No. 1.].

[b10] Beaumont MA, Rannala B (2004). The Bayesian revolution in genetics. Nat. Rev. Genet.

[b11] Berbee ML, Taylor JW (1992). Convergence in ascospore discharge mechanism among pyrenomycete fungi based on 18S ribosomal RNA gene sequence. Mol. Phylogenet. Evol.

[b12] Bidochka MJ, Kamp AM, Lavender TM, Dekoning J, De Croos JNA (2001). Habitat association in two genetic groups of the insect-pathogenic fungus *Metarhizium anisopliae*: uncovering cryptic species?. Appl. Environ. Microbiol.

[b13] Boag B, Yeates GW (1998). Soil nematode biodiversity in terrestrial ecosystems. Biodivers. Conserv.

[b14] Bordallo JJ, Lopez-Llorca LV, Jansson HB, Salinas J, Persmark L, Asensio L (2002). Colonization of plant roots by egg-parasitic and nematode-trapping fungi. New Phytol.

[b15] Brumfield RT, Beerli P, Nickerson DA, Edwards SV (2003). The utility of single nucleotide polymorphisms in inferences of population history. Trends Ecol. Evol.

[b16] Burt AC, Carter DA, Koenig GL, White TJ, Taylor JW (1996). Molecular markers reveal cryptic sex in the human pathogen Coccidioides immitis. Proc. Natl Acad. Sci. USA.

[b17] Cantrell SA, Baez-Félix C (2010). Fungal molecular diversity of a Puerto Rican subtropical hypersaline microbial mat. Fungal Ecol.

[b18] Cantrell SA, Dianese JC, Fell J, Gunde-Cimerman N, Zalar P (2011). Unusual fungal niches. Mycologia.

[b19] Chen R-S, McDonald BA (1996). Sexual reproduction plays a major role in the genetic structure of populations of the fungus *Mycosphaerella graminicola*. Genetics.

[b20] Dietz EJ (1983). Permutation tests for association between two distance matrices. Syst. Biol.

[b21] Douhan GW, Smith ME, Huyrn KL, Westbrook A, Beerli P, Fisher AJ (2008). Multigene analysis suggests ecological speciation in the fungal pathogen *Claviceps purpurea*. Mol. Ecol.

[b22] Duddington CL (1951). The ecology of predaceous fungi I preliminary survey. Trans. Br. Mycol. Soc.

[b23] Duddington CL (1954). Nematode-destroying fungi in agricultural soils. Nature.

[b24] Farrell FC, Jaffee BA, Strong DR (2006). The nematode-trapping fungus *Arthrobotrys oligospora* in soilof the Bodega marine reserve: distribution and dependenceon nematode-parasitized moth larvae. Soil Biol. Biochem.

[b25] Fisher MC, White TJ, Taylor JW (1999). Primers for genotyping single nucleotide polymorphisms and microsatellites in the pathogenic fungus Coccidioides immitis. Mol. Ecol.

[b26] Forche A, Magee PT, Magee BB, May G (2004). Genome-wide single-nucleotide polymorphism map for *Candida albicans*. Eukaryot. Cell.

[b27] Fournier A, Widmer F, Enkerli J (2010). Development of a single-nucleotide polymorphism (SNP) assay for genotyping of *Pandora neoaphidis*. Fungal Biol.

[b28] Fundyga RE, Kuykendall RJ, Lee-Yang W, Lott TJ (2004). Evidence for aneuploidy and recombination in the human commensal yeast *Candida parapsilosis*. Infect. Genet. Evol.

[b29] Gazis R, Rehner S, Chaverri P (2011). Species delimitation in fungal endophyte diversity studies and its implications in ecological and biogeographic inferences. Mol. Ecol.

[b30] Gladieux P, GuÉRin F, Giraud T, Caffier V, Lemaire C, Parisi L (2011). Emergence of novel fungal pathogens by ecological speciation: importance of the reduced viability of immigrants. Mol. Ecol.

[b31] Gray NF (1983). Ecology of nematophagous fungi: *Panagrellus redivivus* as the target organism. Plant Soil.

[b32] Groenewald M, Groenewald JZ, Linde CC, Crous PW (2007). Development of polymorphic microsatellite and single nucleotide polymorphism markers for *Cercospora beticola* (Mycosphaerellaceae). Mol. Ecol. Notes.

[b33] Hallmann J, Davies K, Sikora R, Perry R, Moens M, Starr J (2009). Biological control using microbial pathogens, endophytes and antagonists. Root-knot nematodes.

[b34] Hao Y, Mo MH, Su HY, Zhang KQ (2005). Ecology of aquatic nematode-trapping hyphomycetes in southwestern China. Aquat. Microb. Ecol.

[b35] Hoffmann AA, Parsons PA (1991). Evolutionary genetics and environmental stress.

[b36] Hubisz MJ, Falush D, Stephens M, Pritchard JK (2009). Inferring weak population structure with the assistance of sample group information. Mol. Ecol. Resour.

[b37] Jaffee BA (2004). Wood, nematodes, and the nematode-trapping fungus *Arthrobotrys oligospora*. Soil Biol. Biochem.

[b38] Jaffee BA, Strong DR (2005). Strong bottom-up and weak top-down effects in soil: nematode-parasitized insects and nematode-trapping fungi. Soil Biol. Biochem.

[b39] James TY, Kauff F, Schoch CL, Matheny PB, Hofstetter V, Cox CJ (2006). Reconstructing the early evolution of Fungi using a six-gene phylogeny. Nature.

[b40] Kis-Papo T, Kirzhner V, Wasser SP, Nevo E (2003). Evolution of genomic diversity and sex at extreme environments: fungal life under hypersaline Dead Sea stress. Proc. Natl Acad. Sci. USA.

[b41] Korol AB (1999). Modern perspectives: evolutionary theory and processes.

[b504] Korol A, Preygel I, Preygel S (1994). Recombination Variability and Evolution.

[b42] Kristensen R, Berdal KG, Holst-Jensen A (2007). Simultaneous detection and identification of trichothecene- and moniliformin-producing Fusarium species based on multiplex SNP analysis. J. Appl. Microbiol.

[b43] Kumar D, Singh KP (2006). Assessment of predacity and efficacy of arthrobotrys dactyloides for biological control of root knot disease of tomato. J. Phytopathol.

[b44] Lan L, Xu J (2006). Multiple gene genealogical analyses suggest divergence and recent clonal dispersal in the opportunistic human pathogen *Candida guilliermondii*. Microbiology.

[b45] Li TF, Zhang KQ, Liu XZ (2000). Taxonomy of nematophagous fungi.

[b46] Li Y, Hyde KD, Jeewon R, Cai L, Vijaykrishna D, Zhang KQ (2005). Phylogenetics and evolution of nematode-trapping fungi (Orbiliales) estimated from nuclear and protein coding genes. Mycologia.

[b47] Li L-F, Li T, Zhang Y, Zhao Z-W (2010). Molecular diversity of arbuscular mycorrhizal fungi and their distribution patterns related to host-plants and habitats in a hot and arid ecosystem, southwest China. FEMS Microbiol. Ecol.

[b48] Lopez-Llorca LV, Jansson H-B, Vicente JGM, Salinas J, Schulz BJE, Boyle CJC, Sieber TN (2006). Nematophagous Fungi as Root Endophytes. Microbial root endophytes.

[b49] Meyer SLF, Carta LK, Rehner SA (2005). Morphological variability and molecular phylogeny of the nematophagous fungus *Monacrosporium drechsleri*. Mycologia.

[b50] Mo MH, Chen WM, Su HY, Zhang KQ, Duan CQ, He DM (2006). Heavy metal tolerance of nematode-trapping fungi in lead-polluted soils. Appl. Soil Ecol.

[b51] Morton CO, Mauchline TH, Kerry BR, Hirsch PR (2003). PCR-based DNA fingerprinting indicates host-related genetic variation in the nematophagous fungus *Pochonia chlamydosporia*. Mycol. Res.

[b52] Nevo E (2001). Evolution of genome–phenome diversity under environmental stress. Proc. Natl Acad. Sci. USA.

[b53] Pathak E, El-Borai FE, Campos-Herrera R, Johnson EG, Stuart RJ, Graham JH (2012). Use of real-time PCR to discriminate parasitic and saprophagous behaviour by nematophagous fungi. Fungal Biol.

[b54] Patterson N, Price AL, Reich D (2006). Population structure and eigenanalysis. PLoS Genet.

[b55] Peakall R, Smouse PE (2006). GENALEX 6: genetic analysis in Excel. Population genetic software for teaching and research. Mol. Ecol. Notes.

[b56] Pen-Mouratov S, Hu C, Hindin E, Steinberger Y (2011). Soil microbial activity and a free-living nematode community in the playa and in the sandy biological crust of the Negev Desert. Biol. Fertil. Soils.

[b57] Persson Y, Erland S, Jansson HB (1996). Identification of nematode-trapping fungi using RFLP analysis of the PCR-amplified ITS region of ribosomal DNA. Mycol. Res.

[b58] Pfister D (1995). Two *Arthrobotrys* anamorphs from *Orbilia auricolor*. Mycologia.

[b59] Pritchard JK, Stephens M, Donnelly P (2000). Inference of population structure using multilocus genotype data. Genetics.

[b60] Schmidt AR, Dörfelt H, Perrichot V (2007). Carnivorous fungi from Cretaceous amber. Science.

[b61] Segers R, Butt TM, Carder JH, Keen JN, Kerry BR, Peberdy JF (1999). The subtilisins of fungal pathogens of insects, nematodes and plants: distribution and variation. Mycol. Res.

[b62] Shao Y, Zhang W, Shen J, Zhou LX, Xia HP, Shu WS (2008). Nematodes as indicators of soil recovery in tailings of a lead/zinc mine. Soil Biol. Biochem.

[b63] Sikorski J, Nevo E (2005). Adaptation and incipient sympatric speciation of *Bacillus simplex* under microclimatic contrast at “Evolution Canyons” I and II, Israel. Proc. Natl Acad. Sci. USA.

[b64] Souza EA, Camargo OA, Pinto JM (2010). Sexual recombination in *Colletotrichum lindemuthianum* occurs on a fine scale. Genet. Mol. Res.

[b65] Su H, Hao Ye, Mo M, Zhang K (2007). The ecology of nematode-trapping hyphomycetes in cattle dung from three plateau pastures. Vet. Parasitol.

[b66] Sun S, Heitman J (2011). Is sex necessary?. BMC Biol.

[b67] Swe A, Jeewon R, Pointing S, Hyde K (2009). Diversity and abundance of nematode-trapping fungi from decaying litter in terrestrial, freshwater and mangrove habitats. Biodivers. Conserv.

[b68] Swofford DL (2002). PAUP*: Phylogenetic Analysis Using Parsimony (*and other methods). Version 4.0b10.

[b69] Vandenkoornhuyse P, Leyval C, Bonnin I (2001). High genetic diversity in arbuscular mycorrhizal fungi: evidence for recombination events. Heredity (Edinb.).

[b70] Wachira P, Mibey R, Okoth S, Kimenju J, Kiarie J (2009). Diversity of nematode destroying fungi in Taita Taveta, Kenya. Fungal Ecol.

[b71] Waller PJ (2006). Sustainable nematode parasite control strategies for ruminant livestock by grazing management and biological control. Anim. Feed Sci. Technol.

[b72] Weir BS (1996). Genetic data analysis II.

[b73] West SA, Lively CM, Read AF (1999). A pluralist approach to sex and recombination. J. Evol. Biol.

[b74] Xu J, Vilgalys R, Mitchell TG (2000). Multiple gene genealogies reveal recent dispersion and hybridization in the human pathogenic fungus *Cryptococcus neoformans*. Mol. Ecol.

[b75] Xu J, Guo H, Yang ZL (2007). Single nucleotide polymorphisms in the ectomycorrhizal mushroom *Tricholoma matsutake*. Microbiology.

[b76] Xu J, Sha TAO, Li Y-C, Zhao Z-W, Yang ZL (2008). Recombination and genetic differentiation among natural populations of the ectomycorrhizal mushroom *Tricholoma matsutake* from southwestern China. Mol. Ecol.

[b77] Yang Y, Yang E, An Z, Liu X (2007). Evolution of nematode-trapping cells of predatory fungi of the Orbiliaceae based on evidence from rRNA-encoding DNA and multiprotein sequences. Proc. Natl Acad. Sci. USA.

[b78] Yang E, Xu L, Yang Y, Zhang X, Xiang M, Wang C (2012). Origin and evolution of carnivorism in the Ascomycota (fungi). Proc. Natl Acad. Sci. USA.

[b79] Yeates GW (1979). Soil nematodes in terrestrial ecosystems. J. Nematol.

[b80] Yeates GW, Bongers T (1999). Nematode diversity in agroecosystems. Agric. Ecosyst. Environ.

[b81] Yu ZF, Qiao M, Zhang Y, Zhang KQ (2007). Two new species of *Trichoderma* from Yunnan, China. Antonie Van Leeuwenhoek.

[b82] Zhan J, Pettway RE, McDonald BA (2003). The global genetic structure of the wheat pathogen *Mycosphaerella graminicola* is characterized by high nuclear diversity, low mitochondrial diversity, regular recombination, and gene flow. Fungal Genet. Biol.

[b83] Zhang M, Duan Y, Chen L, Liang C (2002). Effect of agrochemicals and bio-control productions on soil nematode community dynamics. Chin. J. Appl. Ecol.

[b84] Zhang Y, Yu Z-F, Xu J, Zhang K-Q (2011). Divergence and dispersal of the nematode-trapping fungus *Arthrobotrys oligospora* from China. Environ. Microbiol. Rep.

